# Danger of Fly Poisons

**Published:** 1917-04

**Authors:** 


**Danger of Fly Poisons.** The Journal of the Michigan State Medical Society, Mch., reports that it collected in 1916, 36 cases distributed over 14 states, 12 were fatal, 21 recovered, the outcome of 3 was not stated.

